# Serum C3 as an early-warning biomarker for renal pathological progression in DKD

**DOI:** 10.3389/fimmu.2026.1806382

**Published:** 2026-04-15

**Authors:** Donghong Ma, Senle Dai, Tongtong Dai, Dandan Wang, Xue Shan, Jiao Zhang, Jingjing Shi, Yulong Hou, Wenchao Li, Minghao Guo

**Affiliations:** 1 Department of Nephrology, The First Affiliated Hospital of Henan Medical University, Weihui, Henan, China; 2 Xinxiang Key Laboratory of Precise Therapy for Diabetic Kidney Disease, The First Affiliated Hospital of Henan Medical University, Weihui, Henan, China; 3 Department of Renal Pathology, The First Affiliated Hospital of Henan Medical University, Weihui, Henan, China

**Keywords:** diabetic kidney disease, eGFR, IFTA, renal pathology, serum C3

## Abstract

**Objective:**

To investigate the association between serum complement component 3 (C3) levels and renal pathological injury across different estimated glomerular filtration rate (eGFR) strata in patients with diabetic kidney disease (DKD) and evaluate its potential as an early-warning biomarker.

**Methods:**

This retrospective study enrolled 187 DKD patients. Serum C3 levels were measured, and renal biopsies were evaluated by two independent pathologists using a standardized scoring system. Patients were stratified by eGFR (low: <90; high: ≥90 ml/min/1.73m²). The association between serum C3 levels and renal injury was evaluated using multivariable linear regression and binary logistic regression analyses, after adjusting for age, sex, TC, duration of diabetes, HbA1c, albumin and 24-h (24-hour) proteinuria. Restricted cubic spline analysis explored nonlinear relationships in the low eGFR subgroup. Exploratory longitudinal analysis was performed to compare changes in 24-h proteinuria over 1 year of follow-up between patients stratified by serum C3 levels.

**Results:**

Serum C3 levels were negatively correlated with renal C3 deposition and eGFR (ρ = −0.167, *P* = 0.022; ρ = 0.238, *P* = 0.001). In patients with low eGFR, lower C3 levels were consistently associated with higher renal pathology (RP) scoring. After full adjustment, this association remained significant (β = −2.640, *P* = 0.047). Interstitial fibrosis and tubular atrophy and C3 (OR = 0.09, *P* = 0.037), showed significant inverse associations. Restricted cubic spline analysis demonstrated a linear relationship (*P* for overall = 0.031, *P* for nonlinear = 0.079). At 1-year follow-up, exploratory longitudinal analysis showed that patients with lower C3 (<1.10 g/L) showed significantly greater 24-h proteinuria progression (-2869.27 vs. 250.46 mg/24h, *P* = 0.040).

**Conclusion:**

In DKD patients with eGFR <90 ml/min/1.73m², reduced serum C3 levels are associated with specific renal pathological injuries and may serve as a biomarker of disease progression.

## 1 Introduction

Diabetic kidney disease (DKD), one of the most severe microvascular complications of diabetes, has a steadily rising global prevalence. According to the latest data from the International Diabetes Federation, over 463 million adults worldwide have diabetes, a number projected to rise to 700 million by 2045 (1). Approximately 40% of individuals with type 2 diabetes eventually develop DKD, making it the leading cause of end-stage renal disease (ESRD) (2, 3). DKD progresses insidiously and irreversibly. It is clinically characterized by progressive proteinuria and a decline in estimated glomerular filtration rate (eGFR), leading to a doubled risk of cardiovascular events and significantly increased mortality, thereby representing a substantial public health burden (4).

DKD is characterized by classic renal pathology. Core pathological alterations include glomerular basement membrane (GBM) thickening, mesangial matrix expansion, nodular sclerosis (Kimmelstiel-Wilson lesions), accompanied by progressive tubulointerstitial fibrosis and microvascular lesions (5). The management of DKD now represents an integrated approach, which includes lifestyle measures, strict control of blood glucose and pressure, and the use of newer nephroprotective drugs like SGLT-2 inhibitors and GLP-1 receptor agonists. Nevertheless, a proportion of patients still experience relentless decline in renal function, progressing to ESRD in clinical practice. This indicates the existence of unelucidated key pathogenic mechanisms. Recent research has revealed that aberrant activation of the complement system may play a central role in driving inflammatory responses and tissue damage in DKD (6–8).

The complement system is a highly conserved, evolutionarily innate defense mechanism that bridges innate and adaptive immunity (9). Complement component 3 (C3), the central component of the complement cascade, has cleavage fragments (C3a/C3b) that stimulate inflammatory cytokine release and promote mesangial cell proliferation (10, 11). Integrated bioinformatics analysis by Tang et al. demonstrated significant upregulation of C3 in renal tubular tissue, with enrichment in complement and coagulation cascades pathway (12, 13). Moreover, increased complement system activation is observed in both preclinical models of diabetes and clinical samples from patients with DKD. These findings suggest that aberrant complement activation likely contributes to DKD pathogenesis and represents a potential therapeutic target (14, 15).

We systematically investigated the stage-specific characteristics and correlation of serum complement C3 with renal pathological damage, providing a novel theoretical perspective for understanding the stage-dependent mechanisms of DKD. These findings are expected to provide a generalizable objective basis for individualized and precise interventions for DKD.

## 2 Materials and methods

### 2.1 Patient population and study design

**Figure 1:**
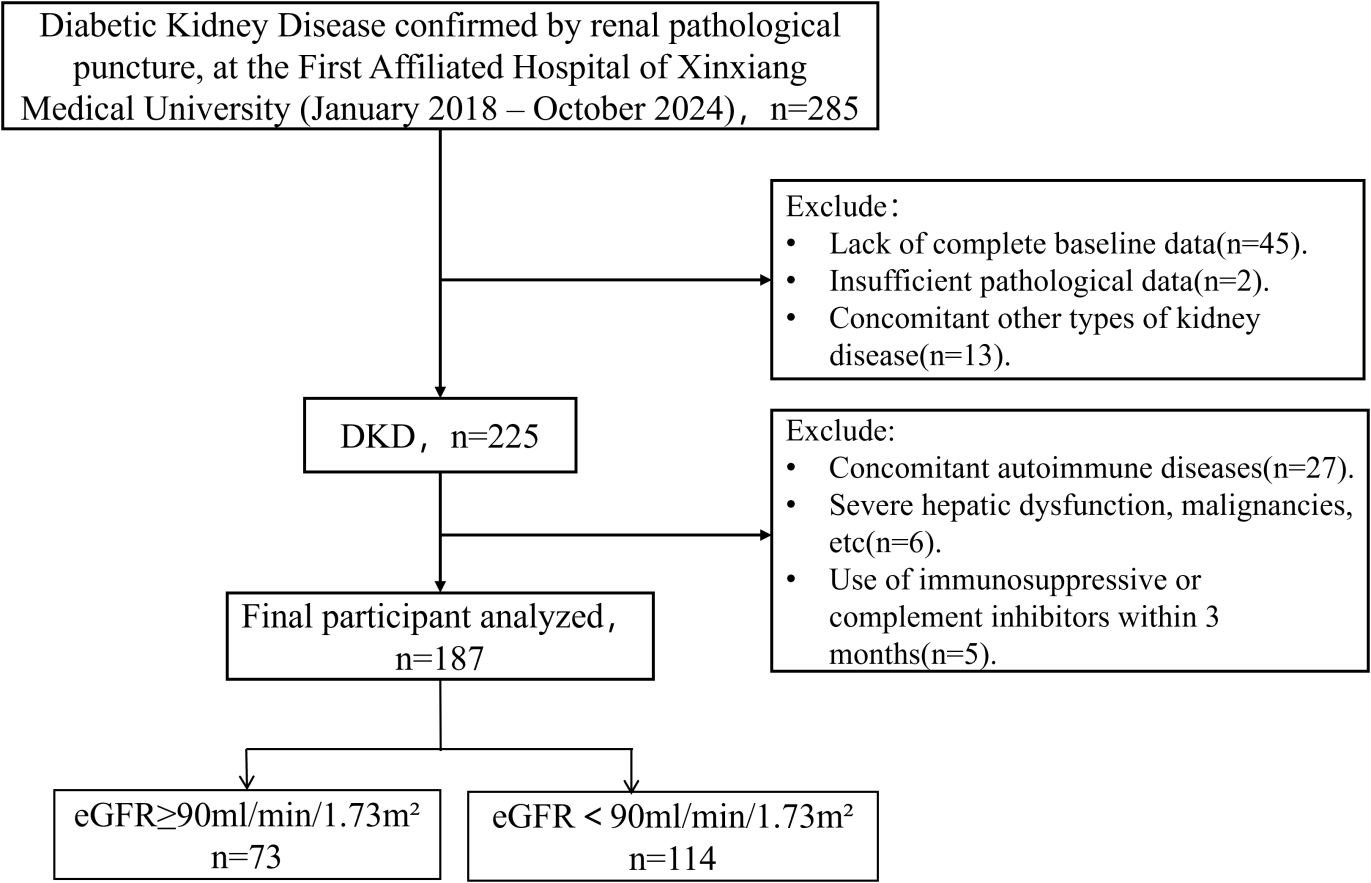
Flow chart of study enrollment. eGFR, estimated glomerular filtration rate.

This retrospective observational study included 187 patients from the First Affiliated Hospital of Henan Medical University who were diagnosed with DKD through renal biopsy and had complete data from 2018 to 2024 (shown in **Figure 1**). Blood and urine samples for laboratory measurements were collected within 48 hours prior to the kidney biopsy during the same admission. Renal tissue samples were evaluated independently by two blinded renal pathologists using a standardized DKD scoring system; any discrepancies were resolved by consensus. This study was approved by the Institutional Review Board. All participants met predefined inclusion/exclusion criteria.

#### 2.1.1 Inclusion criteria

Eligible participants were adults aged 18–80 years, newly diagnosed with diabetic kidney disease, and recruited from the Nephrology Department of the First Affiliated Hospital of Henan Medical University during the period from January 2018 to October 2024.

The clinical indications for renal biopsy in diabetic patients at our center included: (1) acute kidney injury or rapidly progressive renal function decline; (2) abrupt increase in proteinuria; (3) active urinary sediment (e.g., microscopic hematuria or leukocyte casts); (4) absence of diabetic retinopathy or relatively short diabetes duration despite massive proteinuria; and (5) clinical suspicion of concurrent non-diabetic kidney disease. Only patients with biopsy-proven DKD were included in the final analysis.

#### 2.1.2 Exclusion criteria

Initially, 285 inpatients with DKD were screened. Among these, 98 patients were excluded for the following reasons: 1) alternative renal diagnoses (e.g., IgA nephropathy, membranous nephropathy, FSGS); 2) autoimmune conditions affecting kidneys; 3) severe hepatic or oncologic comorbidities; 4) recent use (within 3 months) of immunosuppressive or complement inhibitors; 5) inadequate biopsy samples (< 10 glomeruli or glomerular absence). A total of 187 patients with DKD were included in the analysis.

### 2.2 Data collection

Baseline clinical parameters were retrieved from medical records. Laboratory indices closest to biopsy were systematically recorded. Baseline characteristics included sex, age, and blood pressure (systolic/diastolic). Lipid profiles included serum triglycerides, total cholesterol (TC), high-density lipoprotein cholesterol, and low-density lipoprotein cholesterol. Liver function assessment encompassed albumin (Alb), alanine aminotransferase, and aspartate aminotransferase (AST). Serum components include C3, C4, blood urea, serum creatinine (SCr), erythrocyte sedimentation rate, C-reactive protein (CRP), neutrophil percentage, glycated hemoglobin (HbA1c) and 24-h proteinuria. One-year follow-up data were also collected. Missing data in baseline 24-h proteinuria were handled using multiple imputation. Under the missing at random assumption, 5 imputed datasets were created using the fully conditional specification method. Pooled estimates were calculated based on Rubin’s rules. All assays were performed using standardized automated platforms following manufacturer protocols. The laboratory’s reference range for serum C3 was 0.82–1.8 g/L. eGFR was calculated using the 2009 CKD Epidemiology Collaboration equation.

### 2.3 Histopathologic evaluation

Pathological scoring of renal biopsies was conducted independently by two experienced renal pathologists, following the standardized system for quantifying DKD pathology established in the 2010 JASN publication (5). The comprehensive RP scoring system evaluates six core domains: glomerulosclerosis index (GI, graded by percentage of involved area: Grade 1, 1–25%; Grade 2, 26–50%; Grade 3, >50%), mesangial hypercellularity (MH, scored on a 3-tier scale: mild = 1, moderate = 2, severe = 3), interstitial fibrosis and tubular atrophy (IFTA, stratified as mild = 1, moderate = 2, severe = 3), Kimmelstiel-Wilson nodules (K–W nodules, present = 1, absent = 0), fibrous intimal thickening (FIT, graded on a 4-point scale: none = 0, mild = 1, moderate = 2, severe = 3), and arteriolar hyalinosis (AH, present = 1, absent = 0). Glomerular C3 deposition was semi-quantitatively graded on a 3-point scale:0 = negative, 1 = trace (designated as “±”), 2 = positive (designated as “+”).Scoring divergences were resolved through structured consensus discussion (5).

In the binary logistic regression analysis, outcome variables were defined as follows: Glomerulosclerosis Index was dichotomized as 0 for scores 0–1 and 1 for scores 2-3; Mesangial Hypercellularity was dichotomized as 0 for score 1–2 and 1 for scores 3; Interstitial Fibrosis and Tubular Atrophy was dichotomized as 0 for scores 1–2 and 1 for score 3; Fibrous Intimal Thickening was dichotomized as 0 for scores 0–2 and 1 for score 3.

### 2.4 Stratification of eGFR

In this study, based on the KDIGO Chronic Kidney Disease (CKD) staging criteria (16), patients were stratified by eGFR using a cutoff of 90 mL/min/1.73m². This stratification aimed to distinguish between two disease stages with fundamental differences: normal renal function with evidence of kidney damage (CKD G1 stage) and definite decline in renal function (CKD G2 stage and beyond). This dichotomy was chosen to compare patients with normal or near-normal kidney function against those with any degree of renal impairment, a distinction that is readily applicable in clinical practice for risk stratification and treatment decisions.

### 2.5 Statistical analysis

Quantitative data were assessed for normality using the Shapiro-Wilk test. Normally distributed continuous variables were expressed as mean ± standard deviation and compared between groups using independent sample t-tests for two groups or one-way ANOVA for multiple groups. Non-normally distributed data were presented as median (interquartile range) and analyzed using Mann-Whitney U test (two groups) or Kruskal- Wallis test (multiple groups). Categorical variables were presented as number (percentage) and compared using chi-square tests.

Linear regression analysis was performed to examine the association between serum C3 levels and RP scores. To assess the robustness of the regression coefficients and to account for the non-normal distribution of residuals, we performed bootstrap resampling with 1,000 replications. Bias-corrected and accelerated (BCa) 95% confidence intervals were calculated for all estimates. Multiple regression models were constructed with progressive adjustment. Results were expressed as β coefficients with (BCa) 95% confidence intervals (CI) to models 1-3. Model 4 was expressed as β coefficients with 95% confidence intervals (CI) due to the reduced subgroup sample size (n=77). As a sensitivity analysis, the composite RP score was dichotomized at the median (score >10 vs ≤10) and analyzed using binary logistic regression (Model 5). Results are presented as odds ratios (OR) with 95% confidence intervals (CI) (n = 114). The selection of confounding factors was determined based on clinical significance.

Based on the KDIGO chronic kidney disease staging system, the eGFR threshold of 90 ml/min/1.73 m² was used as the key cutoff for subgroup stratification. Interaction *P*-values were calculated to assess whether the association between serum C3 and RP scores differed significantly between eGFR subgroups.

Restricted cubic splines (RCS) analysis with three knots was employed to evaluate potential non-linear relationships between serum C3 levels and RP scores in participants with eGFR < 90 ml/min/1.73m (2). The overall association and test for non-linearity were assessed, with *P*-values reported for overall significance and deviation from linearity.

The relationships of serum C3 levels with eGFR and renal C3 deposition were assessed using Spearman’s rank correlation test. Individual pathological components including GI, MH, Interstitial fibrosis and tubular atrophy, K–W nodules, FIT and AH were analyzed separately using binary logistic regression adjusted for age, sex, TC, duration of diabetes, HbA1c, albumin and 24-h proteinuria.

To assess potential attrition bias, we compared baseline demographic, clinical, and laboratory characteristics between patients with and without 1-year follow-up data. An exploratory longitudinal analysis of C3 and 24-h proteinuria was performed.

All statistical processes were conducted using the R software package (V.4.0; The R Foundation; http://www.R-project.org) and SPSS 27. A two-tailed *P*-value of less than 0.05 was considered statistically significant.

## 3 Results

### 3.1 Baseline characteristics of the participants

A total of 187 patients with DKD were included, with a mean age of 53.64 ± 10.48 years, of whom 68.45% were male. Participants were stratified by eGFR into two groups: the high eGFR group (≥ 90 mL/min/1.73 m², n = 73) and the low eGFR group (< 90 mL/min/1.73 m², n = 114). The median eGFR was significantly higher in the high eGFR group than in the low eGFR group (118.74 vs. 44.27 mL/min/1.73 m², *P* < 0.001). No significant difference was observed in duration of diabetes and diabetic retinopathy between the low and high eGFR groups (χ² = 1.50, *P* = 0.221). The distribution of RAAS inhibitor and SGLT2 inhibitor use differed significantly between the two groups (χ² = 23.92, *P* < 0.001; χ²=10.40, *P* = 0.001). The low eGFR group was older than the high eGFR group (55.48 ± 10.59 vs. 50.75 ± 9.70, *P* < 0.01), and erythrocyte sedimentation rates (28.00 vs. 18.00, *P* = 0.01). Renal function markers also differed substantially: the low eGFR group demonstrated significantly higher blood urea (10.33 vs. 6.28 mmol/L, *P* < 0.001) and SCr (156.50 vs. 67.00 μmol/L, *P* < 0.001), as well as lower Alb levels (35.90 vs. 38.20 g/L, *P* = 0.032). They also had lower triglycerides (1.61 vs. 2.07 mmol/L, *P* < 0.01), alanine aminotransferase (16.00 vs. 20.00 U/L, *P* < 0.01) and HbA1c (6.73% vs. 7.26%, *P* < 0.01). Regarding serum components, the low eGFR group had lower C3 complement (1.14 vs. 1.06 g/L, *P* = 0.022) and marginally higher C4 complement (0.31 vs. 0.27 g/L, *P* = 0.069) compared to the higher eGFR group. Advanced CKD stages were more frequent in the low eGFR group (*P* < 0.01), who also exhibited higher RP scores (10.50 ± 2.76 vs. 9.79 ± 2.81, *P* < 0.01). Both groups demonstrated high baseline levels of 24-h proteinuria (4130.48 vs. 4240.24). No statistically significant differences were observed in sex distribution, SBP, CRP, TC, HDL, LDL, AST levels, 24-h proteinuria or RP Score. (**Table 1**).

**Table 1:** Baseline characteristics of participants by eGFR levels.

Variables	Total (n = 187)	High eGFR^a^ (n = 73)	Low eGFR^b^ (n = 114)	Statistic	*P* Value
Anthropometric data					
Age (years)	53.64 ± 10.48	50.75 ± 9.70	55.48 ± 10.59	t = −3.078	0.002
Male, n (%)	128 (68.45)	51 (69.86)	77 (67.54)	χ² = 0.111	0.750
SBP (mmHg)	150.47 ± 21.64	147.90 ± 18.88	152.02 ± 23.11	t=-1.16	0.249
DBP (mmHg)	89.22 ± 12.66	91.86 ± 12.49	87.63 ± 12.56	t=2.05	0.042
Duration of Diabetes(years)	8.00 (3.00, 12.50)	7.00 (2.00, 13.00)	10.00 (5.00, 12.00)	Z=-1.81	0.070
Diabetic retinopathy, n (%)	92 (49.20)	40 (54.79)	52 (45.61)	χ² = 1.50	0.221
Drug therapy					
RAAS inhibitors, n (%)	110 (58.82)	59 (80.82)	51 (44.74)	χ² = 23.92	< 0.001
SGLT2 inhibitors, n (%)	98 (52.41)	49 (67.12)	49 (42.98)	χ² =10.40	0.001
Biochemical parameters					
ESR (mm/H)	24.0 (10.00, 40.00)	18.00 (9.50, 33.25)	28.00 (12.00, 48.00)	Z = 2.574	0.01
CRP (mg/L)	1.37 (0.57, 4.11)	1.14 (0.52, 3.82)	1.46 (0.60, 5.06)	Z = 0.940	0.347
TC (mmol/L)	4.93 (4.11, 6.28)	4.95 (4.01, 6.34)	4.87 (4.12, 6.25)	Z=-0.35	0.728
HDL (mmol/L)	1.28 ± 0.48	1.27 ± 0.50	1.28 ± 0.46	t = −0.149	0.881
LDL (mmol/L)	2.95 ± 1.24	3.02 ± 1.36	2.90 ± 1.16	t = −0.695	0.488
TG (mmol/L)	1.81 (1.27, 2.55)	2.07 (1.45, 2.77)	1.61 (1.23, 2.28)	Z = −2.656	0.008
ALT (U/L)	17.00 (12.00, 26.50)	20.00 (14.00, 30.00)	16.00 (10.00, 25.00)	Z = −2.705	0.007
AST (U/L)	19.00 (14.00, 23.00)	19.00 (16.00, 24.00)	18.00 (14.00, 23.00)	Z = −1.057	0.291
Alb (g/L)	36.50 (30.90, 41.10)	38.20 (32.20, 42.10)	35.90 (29.57, 40.48)	Z=-2.14	0.032
BU (µmol/L)	8.23 (6.30,11.77)	6.28 (5.34,7.81)	10.33 (7.99,14.67)	Z = 8.411	< 0.001
SCr (µmol/L)	108.40 (72.35, 175.15)	67.00 (58.70, 77.90)	156.50 (114.45, 209.88)	Z = 11.203	< 0.001
HbAlc (%)	7.03 (6.00, 7.99)	7.26 (6.40, 8.32)	6.73 (5.69, 7.62)	Z=-2.78	0.006
C3 (g/L)	1.09 ± 0.24	1.14 ± 0.25	1.06 ± 0.23	t=2.30	0.022
C4 (g/L)	0.30 (0.24, 0.38)	0.27 (0.24, 0.37)	0.31 (0.25, 0.39)	Z = 1.818	0.069
CKD stage, n (%)				χ² = 130.16	< 0.01
1	73 (39.04)	73 (81.11)	0 (0.00)		
2	37 (19.79)	3 (3.33)	34 (35.05)		
3	46 (24.60)	9 (10.00)	37 (38.14)		
4/5	31 (16.58)	5 (5.56)	26 (26.80)		
eGFR(mL/min/1.73m²)	71.04 (40.78, 113.78)	118.74 (109.66, 127.47)	44.27 (28.44, 67.78)	Z=-11.52	<.001
24-h proteinuria (mg/24h)	4179.89 (1937.42, 7253.24)	4130.48 (2623.98, 6891.16)	4240.24 (1678.26, 7348.04)	Z=-0.06	0.953
Renal pathology score	10.22 ± 2.79	9.79 ± 2.81	10.50 ± 2.76	t = −1.693	0.092

eGFR^a^ ≥ 90 mL/min/1.73m²; eGFR^b^ < 90 mL/min/1.73m².

Alb, albumin; ALT, alanine aminotransferase; AST, aspartate aminotransferase; BU, blood urea; C3, complement component 3; C4, complement component 4; CKD, chronic kidney disease; CRP, C-reactive protein; SBP, systolic blood pressure; DBP, diastolic blood pressure; ESR, erythrocyte sedimentation rate; HbA1c, glycated hemoglobin; HDL, high-density lipoprotein cholesterol; LDL, low-density lipoprotein cholesterol; SCr, serum creatinine; TC, total cholesterol; TG, triglycerides.

### 3.2 Correlation between eGFR and serum C3 levels

**Figure 2:**
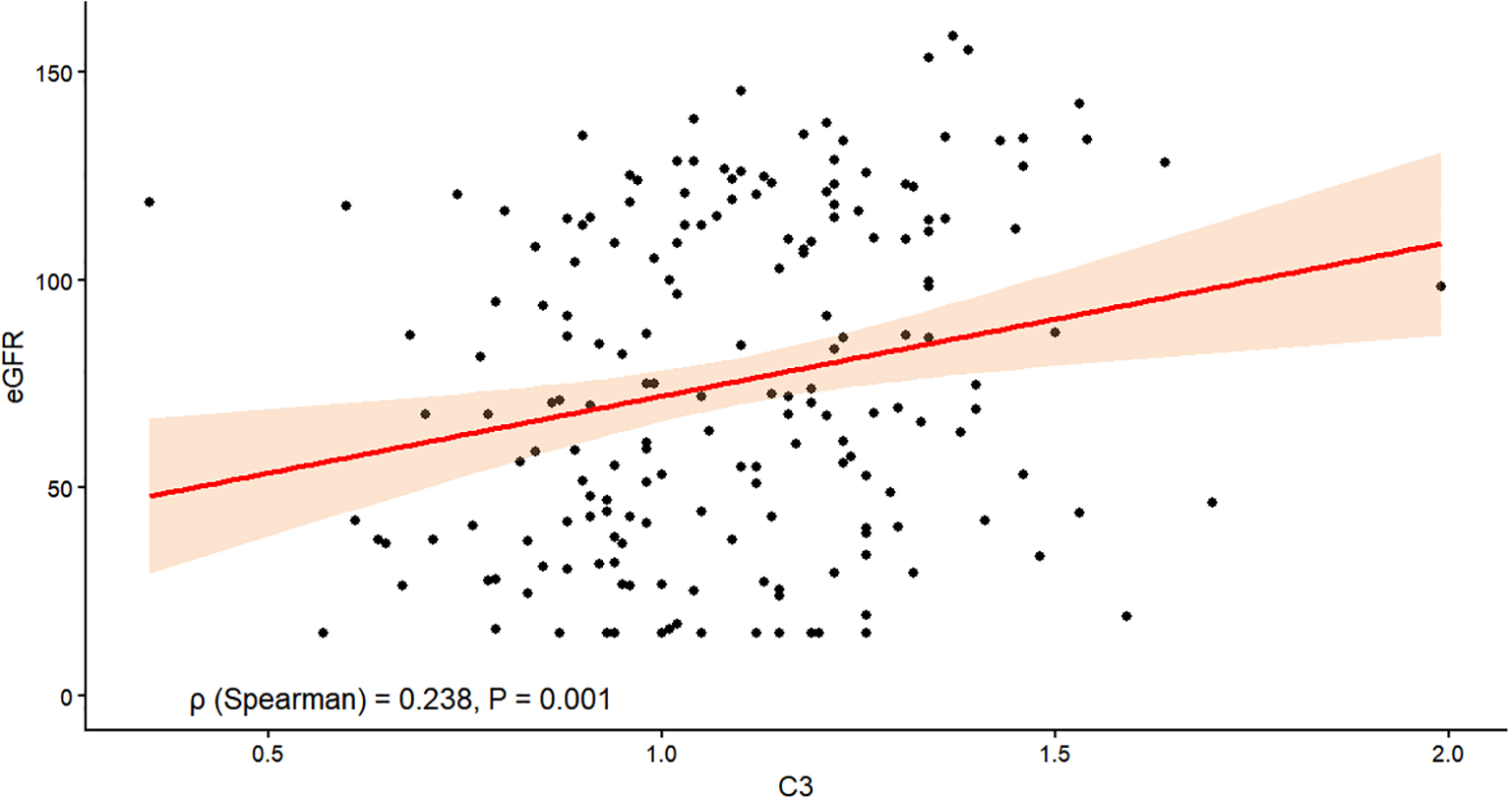
Correlation analysis between serum C3 and eGFR. Scatter plot showing the relationship between serum C3 levels (x-axis) and eGFR (y-axis). The Spearman correlation coefficient was ρ= 0.238 (*P* = 0.001).

As shown in **Figure 2**, correlation analysis revealed a significant positive association between serum C3 levels and eGFR in patients with DKD. The Spearman correlation coefficient was ρ= 0.238 (*P* = 0.001). This finding demonstrated a statistically significant positive relationship between these two variables.

### 3.3 Pathological features of DKD stages II-IV

**Figure 3:**
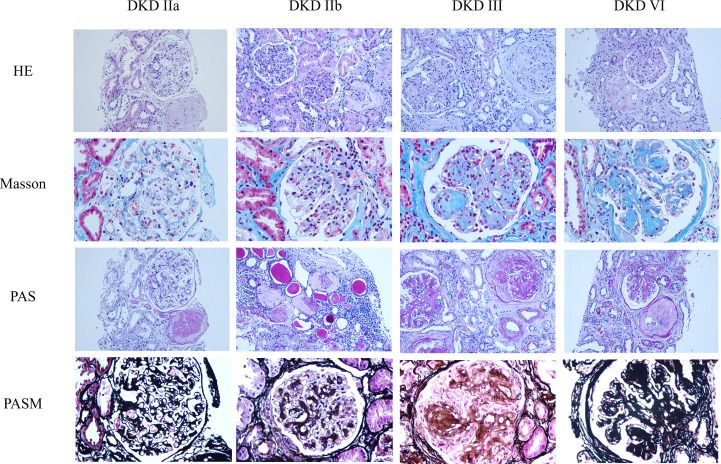
Representative kidney pathology images in diabetic kidney disease. DKD pathological stages are classified according to the RPS system: Class I: isolated glomerular basement membrane thickening; Class II: mesangial expansion (IIa: mild > 25%, area < capillary lumen; IIb: severe > 25%, area > lumen); Class III: nodular sclerosis with Kimmelstiel-Wilson nodules; Class IV: > 50% global glomerulosclerosis with diabetic evidence. HE × 200; Masson × 400; PAS × 200; PASM × 400.

DKD exhibits distinct pathological features across stages, from isolated GBM thickening (Class I) to mesangial expansion (Class II a/b), nodular sclerosis (Class III) and diffuse glomerular scarring (Class IV). Class II severity is quantified by mesangial-to-capillary area ratios, Class III by K-W nodules and Class IV is defined by > 50% global sclerosis with confirmation of diabetic etiology (5). These objective, quantifiable pathological hallmarks provide a critical framework for staging disease severity and confirming the diabetic etiology of the lesions (5) (shown in **Figure 3**).

### 3.4 Correlation analysis between serum C3 and renal C3 deposition

**Figure 4:**
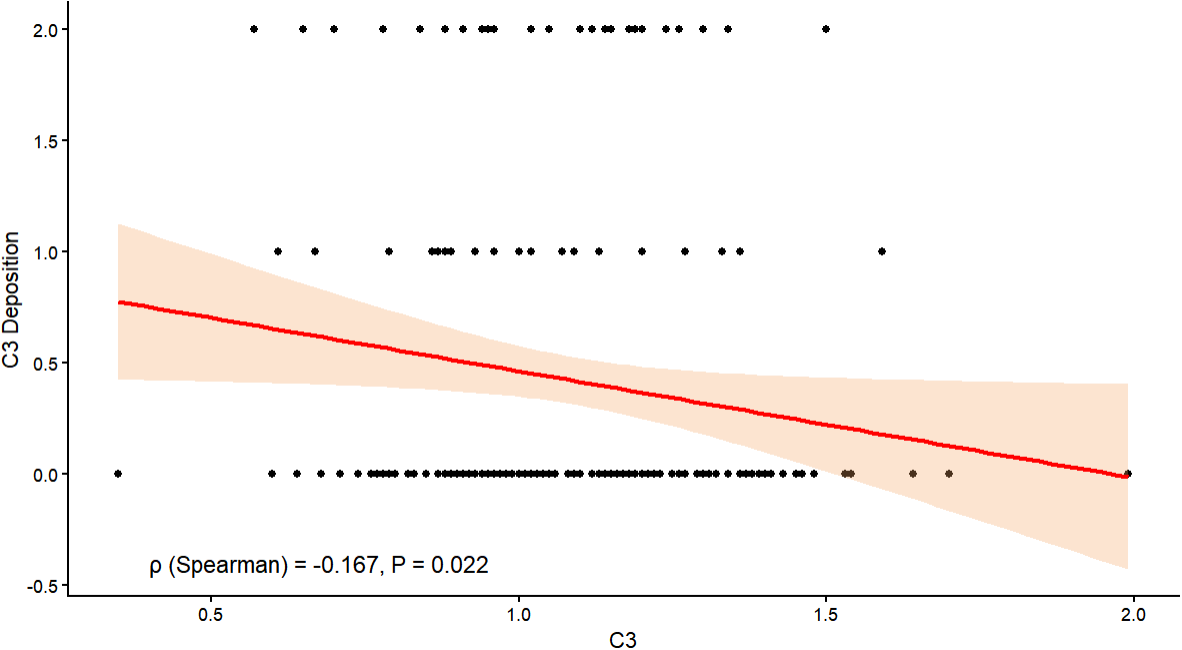
Correlation analysis between serum C3 and renal C3 deposition. Scatter plot showing the relationship between serum C3 levels (x-axis) and renal C3 deposition (y-axis). The Spearman correlation coefficient was ρ= −0.167 (*P* = 0.022).

As shown in **Figure 4**, correlation analysis revealed a significant negative association between serum C3 levels and renal C3 deposition in patients with DKD. The Spearman correlation coefficient was ρ= −0.167 (*P* = 0.022), indicating a statistically significant inverse relationship between these two variables.

### 3.5 Association between serum C3 and RP score by eGFR

**Table 2** shows the relationship between C3 levels and RP scores that varied significantly by eGFR status: in patients with eGFR < 90 ml/min/1.73m (2), C3 demonstrated a consistent inverse association with RP scores across all models, with unadjusted analysis showing a β coefficient of −2.592 (BCa 95% CI: −4.651 to −0.434, *P* = 0.017), indicating that higher C3 levels were associated with lower (better) RP scores. After adjustment for age, sex and TC in Model 2, the association remained virtually unchanged (β = −2.720, BCa 95% CI: -4.948 to -0.589, *P* = 0.025), and persisted after full adjustment for age, sex, TC, duration of diabetes, HbA1c, albumin and 24-h proteinuria in Model 3 (β = −2.640, BCa 95% CI: −5.222 to −0.301, *P* = 0.047). In subgroup sensitivity analysis of patients with eGFR < 60 mL/min/1.73 m², C3 remained negatively associated with the RP scores in Model 4 after full adjustment for age, sex, TC, diabetes duration, HbA1c, albumin and 24-h proteinuria (β = −3.49, 95% CI: -6.26 to -0.72, *P* = 0.017). In a sensitivity analysis of patients with eGFR < 90 mL/min/1.73 m², after full adjustment for age, sex, TC, duration of diabetes, HbA1c, albumin, and 24-h proteinuria, C3 showed a significant negative association with the dichotomized renal pathology score (≤10 defined as 0, >10 defined as 1) in Model 5 (OR = 0.10, 95% CI: 0.01 to 0.95, *P* = 0.045).

**Table 2:** Association between serum C3 and renal pathology score by eGFR levels.

Model^a^	Low eGFR^b^ β, 95%CI		*P* value	High eGFR^c^ β, 95%CI	*P* value	*P* for interaction
Model 1	-2.592 (-4.651, -0.434)		0.017	1.560 (-1.015, 4.486)	0.244	0.029
Model 2	-2.720 (-4.948, -0.589)		0.025	1.241 (-1.172, 4.323)	0.393	0.023
Model 3	-2.640 (-5.222, -0.301)		0.047	1.742 (-0.925, 5.226)	0.196	0.044
Subgroup analysis						
		eGFR < 60 mL/min/1.73m²(n=77) β, 95%CI			*P* value	
Model 4		-3.49 (-6.26 ~ -0.72)			0.017	
Sensitivity Analysis						
		eGFR < 90 mL/min/1.73m²(n=114) OR, 95%CI			*P* value	
Model 5		0.10 (0.01 ~ 0.95)			0.045	

Model 1 was not adjusted; Model 2 was adjusted for age, sex and TC; Model 3, 4 and 5 were adjusted for age, sex, TC, duration of diabetes, HbA1c, albumin and 24-h proteinuria.

Bootstrap standard errors (1000 resamples, BCa 95% CI) were applied to account for non-normality of residuals. Bootstrap CIs were applied to Models 1–3; Model 4 reports conventional OLS estimates due to the reduced subgroup sample size (n=77). Model 5: binary logistic regression with RP score dichotomized at median (>10 vs. ≤10).

eGFR^b^ < 90 mL/min/1.73m²; eGFR^c^ ≥ 90 mL/min/1.73m².

C3, complement component 3; CI, confidence interval; TC, total cholesterol; eGFR, estimated glomerular filtration rate; SBP, systolic blood pressure.

Conversely, in patients with eGFR ≥ 90 ml/min/1.73m (2), no significant association was observed between C3 and RP scores in any model: unadjusted analysis β = 1.560 (BCa 95% CI: −1.015 to 4.486, *P* = 0.244), age, sex and TC adjusted β = 1.49 (BCa 95% CI: −1.172 to 4.323, *P* = 0.393), and fully adjusted β = 1.742 (BCa 95% CI: −0.925 to 5.226, *P* = 0.196).

The interaction between C3 and eGFR status was statistically significant across all models between the two eGFR groups (*P* for interaction: 0.029, 0.023, 0.044 for Models 1, 2 and 3, respectively).

### 3.6 RCS analysis of serum C3 and RP score in patients with DKD and eGFR < 90 ml/min/1.73m²

**Figure 5:**
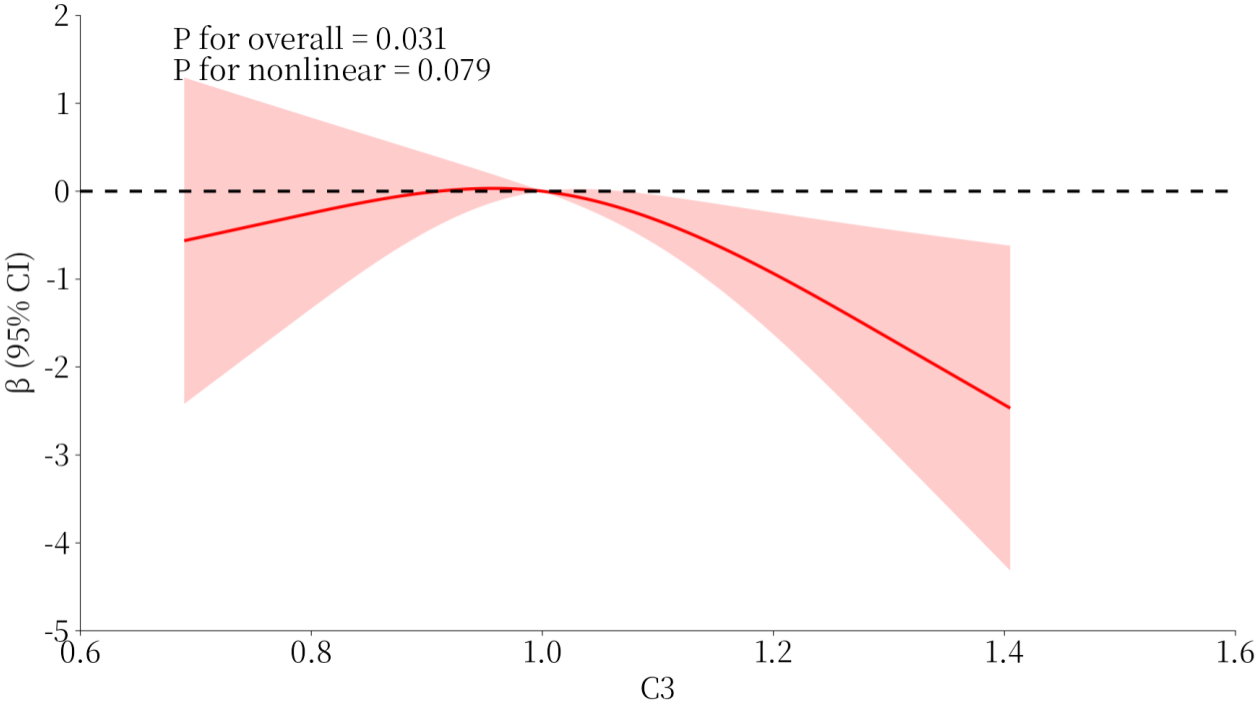
Restricted cubic splines curve of serum C3 and renal pathology score in in patients with eGFR < 90 mL/min/1.73m². The curve shows the non-linear association between plasma C3 levels (x-axis) and renal pathology score (y-axis, expressed as β coefficient with 95% CI). The red line represents the spline curve with 95% CI (pink shaded area). The horizontal dashed line at β = 0 serves as the reference. *P* for overall = 0.031, *P* for nonlinear = 0.079. CI, confidence interval; eGFR, estimated glomerular filtration rate.

As shown in **Figure 5**, RCS curve analysis demonstrated a significant linear relationship between serum C3 levels and RP scores in patients with DKD and eGFR < 90 ml/min/1.73 m². The overall association was statistically significant (*P* for overall = 0.031), while the test for non-linearity yielded *P* = 0.079, suggesting that the relationship did not significantly deviate from non-linearity.

### 3.7 Association between serum C3 and the specific pathological components in patients with DKD and eGFR < 90 ml/min/1.73m²

As shown in **Table 3**, in patients with DKD and eGFR < 90 ml/min/1.73m², the analysis of individual RP components revealed varying associations with C3 levels after adjustment for age, sex, TC, duration of diabetes, HbA1c, albumin and 24-h proteinuria.

**Table 3:** Association between serum C3 and renal pathology score in patients with eGFR <90 mL/min/1.73m².

Outcomes	OR, 95% CI	*P* value
Glomerulosclerosis Index	0.90 (0.12 ~ 6.70)	0.917
Mesangial Hypercellularity	0.41 (0.05 ~ 3.35)	0.403
IFTA	0.09 (0.01 ~ 0.87)	0.037
K−W nodules	0.25 (0.03 ~ 2.12)	0.205
Fibrous Intimal Thickening	0.18 (0.02 ~ 1.59)	0.123
Arteriolar Hyalinosis	0.38 (0.04 ~ 3.43)	0.388

Model was adjusted for age, sex, TC, duration of diabetes, HbA1c, albumin and 24-h proteinuria.

C3, complement component 3; OR: Odds Ratio, CI, confidence interval; eGFR, estimated glomerular filtration rate; IFTA, interstitial fibrosis and tubular atrophy; K-W nodules, Kimmelstiel-Wilson nodules; TC, total cholesterol.

Among the specific pathological components, IFTA showed a significant inverse association with C3 (OR = 0.09, 95% CI: 0.01 to 0.87, *P* = 0.037), suggesting that higher C3 levels were associated with less severe tubulointerstitial damage. In contrast, GI showed no significant association with C3 (*P* = 0.917), while MH demonstrated a non-significant trend toward inverse association (*P* = 0.403). K−W Nodules showed no significant association with C3 levels (*P* = 0.205), FIT was not significantly correlated with C3 levels (*P* = 0.123), and AH also showed no statistically significant associations with C3 levels (*P* = 0.388).

### 3.8 Exploratory longitudinal analysis

#### 3.8.1 Baseline characteristics of patients with and without 1-year follow-up

**Table 4** compares baseline characteristics between patients with (n=31) and without (n=156) 1-year follow-up. Patients with follow-up exhibited significantly higher SCr, lower eGFR, and more severe Renal Pathology Score at baseline(*P*<0.001). In contrast, Demographic characteristics (age, sex), clinical parameters (SBP, DBP, diabetes duration, diabetic retinopathy), and laboratory findings (HbA1c, 24-h proteinuria, CRP, TC, ALT, AST, Alb) were all comparable between the two groups(*P*>0.05).

**Table 4:** Baseline characteristics of patients with and without 1-year follow-up.

Variables	Total (n = 187)	With follow-up (n = 31)	Without follow-up (n = 156)	Statistic	*P*
Anthropometric data					
Age (years)	53.64 ± 10.48	52.74 ± 9.83	53.81 ± 10.63	t=-0.52	0.604
Male, n (%)	128 (68.45)	20 (64.52)	108 (69.23)	χ²=0.27	0.606
SBP (mmHg)	150.47 ± 21.64	146.53 ± 19.03	151.40 ± 22.18	t=-1.11	0.269
DBP (mmHg)	89.22 ± 12.66	88.37 ± 11.13	89.43 ± 13.03	t=-0.41	0.682
Duration Of Diabetes, (years)	8.00 (3.00, 12.50)	6.00 (3.50, 10.00)	9.50 (3.00, 13.00)	Z=-0.84	0.400
Diabetic retinopathy, n (%)	92 (49.20)	13 (41.94)	79 (50.64)	χ²=0.78	0.376
Drug therapy					
RAAS inhibitors, n (%)	110 (58.82)	13 (41.94)	97 (62.18)	χ²=4.38	0.036
SGLT2 inhibitors, n (%)	98 (52.41)	12 (38.71)	86 (55.13)	χ²=2.79	0.095
Biochemical parameters					
CRP (mg/L)	1.38 (0.56, 4.15)	0.88 (0.45, 3.50)	1.39 (0.60, 4.72)	Z=-0.94	0.345
TC (mmol/L)	4.93 (4.11, 6.28)	4.80 (4.11, 6.29)	4.95 (4.12, 6.27)	Z=-0.45	0.651
ALT (U/L)	17.00(12.00, 26.50)	16.00 (11.50, 21.50)	18.00(12.00, 27.00)	Z=-0.98	0.328
AST (U/L)	19.00(14.00, 23.00)	18.00 (13.00, 21.50)	19.00(14.00, 23.25)	Z=-0.77	0.442
Alb (g/L)	36.50 (30.90, 41.10)	36.40 (32.10, 40.05)	36.75 (30.45, 41.12)	Z=-0.25	0.801
SCr (µmol/L)	108.40(72.35, 175.15)	161.00(114.90, 192.60)	95.00(68.58, 164.30)	Z=-3.37	<.001
HbA1c (%)	7.03 (6.00, 7.99)	6.76 (5.53, 7.60)	7.11 (6.00, 8.00)	Z=-1.11	0.268
24-h proteinuria (mg/24h)	4179.89 (1937.42, 7253.24)	4572.27 (1737.71, 7277.84)	4130.48 (2170.76, 7182.03)	Z=-0.04	0.965
eGFR(mL/min/1.73m²)	71.04 (40.78, 113.78)	47.14 (35.64, 67.60)	83.96(42.74,116.8)	Z=-3.48	<.001
Renal Pathology Score	10.00 (8.00, 12.00)	11.00 (10.50, 13.00)	10.00 (8.00, 12.00)	Z=-3.44	<.001

Alb, albumin; ALT, alanine aminotransferase; AST, aspartate aminotransferase; BU, blood urea; CRP, C-reactive protein; SBP, systolic blood pressure; DBP, diastolic blood pressure; HbA1c, glycated hemoglobin; HDL, high-density lipoprotein cholesterol; LDL, low-density lipoprotein cholesterol; SCr, serum creatinine; TC, total cholesterol; TG, triglycerides.

The reasons for the low follow-up rate were as follows: transfer to local hospitals (n=62, 39.7%), change of contact information (n=16,10.3%), incomplete clinical examinations (n=54, 34.6%), initiation of renal replacement therapy (n=19,12.2%) and death (n=5, 3.2%).

#### 3.8.2 Comparison of 24-h proteinuria change at 1-year follow-up between groups by median serum C3 level

We assessed changes in 24-h proteinuria, a key marker of glomerular filtration barrier impairment in DKD, at 1-year follow-up. As shown in **Table 5**, 31 patients with DKD who completed a 1-year follow-up were grouped according to the median serum C3 level: 20 (64.5%) had C3 < 1.10 g/L, and 11 (35.5%) had C3 ≥ 1.10 g/L. Patients in the lower C3 group showed a significantly greater increase in 24-h proteinuria at 1-year follow-up compared with the higher C3 group (−2869.27vs. 250.46, *P* = 0.040). There were no significant differences in diabetes duration, diabetic retinopathy, or the use of RAAS inhibitors and SGLT2 inhibitors between the two groups. No significant between-group differences were found in age, systolic blood pressure, diastolic blood pressure, Alb, TC, AST, HbA1c or 24-h proteinuria (all *P* > 0.05). However, due to the small sample size and high rate of loss to follow-up, these findings should be interpreted with caution.

**Table 5:** Comparison of 24-h proteinuria changes at 1-year follow-up by median serum C3 in patients with eGFR < 90 mL/min/1.73m².

Variables	Total (n = 31)	Low serum C3^a^ (n = 20)	Higher serum C3^b^ (n = 11)	Statistic	*P* Value
Anthropometric data					
Age (years)	52.74 ± 9.83	52.70 ± 10.28	52.82 ± 9.42	t=-0.03	0.975
Male, n (%)	20 (64.52)	14 (70.00)	6 (54.55)	-	0.452
SBP (mmHg)	146.53 ± 19.03	147.05 ± 19.58	145.50 ± 18.85	t = 0.21	0.838
DBP (mmHg)	88.37 ± 11.13	87.60 ± 10.23	89.90 ± 13.20	t = −0.53	0.602
Duration of Diabetes (years)	6.00 (3.50, 10.00)	6.50 (2.75, 10.00)	6.00 (4.50, 9.00)	Z=-0.29	0.771
Diabetic retinopathy, n (%)	13 (41.94)	8 (40.00)	5 (45.45)	-	1.000
Drug therapy					
RAAS inhibitors, n (%)	13 (41.94)	10 (50.00)	3 (27.27)	-	0.275
SGLT2 inhibitors, n (%)	12 (38.71)	6 (30.00)	6 (54.55)	-	0.255
Biochemical parameters					
AST (U/L)	18.00 (13.00, 21.50)	16.00 (12.00, 20.00)	19.00 (17.00, 29.00)	Z=-1.47	0.142
Alb (g/L)	35.87 ± 6.24	35.89 ± 5.79	35.85 ± 7.29	t=0.02	0.985
SCr (μmol/L)	165.82 ± 75.92	183.86 ± 86.17	133.03 ± 36.73	t=1.85	0.074
24-h proteinuria (mg/24h)	3852.44 (1695.75, 6103.75)	3697.30 (1528.92, 5697.32)	4805.33 (2097.54, 7534.03)	Z=-0.72	0.471
24-h proteinuria change^c^ (mg/24h)	-1834.30 (-5189.91, 255.41)	-2869.27 (-5611.54, -1048.38)	250.46 (-2587.80, 1976.28)	Z=-2.06	0.040

Continuous variables with a normal distribution are presented as Mean ± SD, and comparisons between the two groups were performed using an independent samples t-test. Continuous data with a skewed distribution are presented as M (Q_1_, Q_3_), and an independent samples rank-sum test was used for inter-group comparisons. A P-value < 0.05 was considered statistically significant.

C3^a^ < 1.10 g/L; C3^b^ ≥ 1.10 g/L.

The 24-hour proteinuria change^c^ was calculated as baseline 24-hour proteinuria minus 24-hour proteinuria at re-examination.

Alb, albumin; AST, aspartate aminotransferase; C3, complement component 3; SBP, systolic blood pressure; DBP, diastolic blood pressure; eGFR, estimated glomerular filtration rate; SCr, serum creatinine; TC, total cholesterol.

## 4 Discussion

In this study of 187 patients with DKD, we investigated the relationship between serum C3 levels and renal pathological damage across different eGFR strata, aiming to evaluate its potential as an early-warning biomarker for disease progression. Our findings demonstrated that in patients with eGFR < 90 mL/min/1.73 m², lower serum C3 levels were significantly associated with more severe renal pathological injury, and showed a significant interaction with the eGFR ≥ 90 mL/min/1.73m² subgroup. Subgroup analysis of pathological injury variables revealed that serum complement C3 was significantly and inversely correlated with IFTA severity in patients with lower eGFR. Furthermore, RCS curve analysis demonstrated a significant linear relationship between serum C3 levels and RP scores in this subgroup. Follow-up analysis showed that patients in the lower C3 group had a significantly greater increase in 24-h proteinuria change at 1-year follow-up compared with the higher C3 group, suggesting that patients with lower serum C3 represent a high-risk population for DKD progression and adverse renal prognosis. In summary, our findings suggest that serum C3 monitoring could serve as a non-invasive marker for assessing tubulointerstitial injury in patients with DKD and reduced eGFR. Renal function and urinary protein dynamics should be closely monitored in this high-risk subgroup during clinical follow-up.

Patients in the eGFR ≥ 90 mL/min/1.73m² group (CKD G1 stage) are at a stage of compensatory hyperfiltration and early renal injury. Essentially, the kidney is in a “compensatory” or “subclinical injury” phase. Once eGFR drops below 90 mL/min/1.73m², the disease progresses to a rapid progression stage dominated by structural damage. Sustained intraglomerular hypertension results in endothelial cell injury, GBM thickening and mesangial dissolution and expansion, ultimately triggering progressive glomerulosclerosis and renal interstitial fibrosis (17). Our study found that, in patients at this stage, serum complement C3 levels were inversely correlated with the severity of IFTA. This finding suggests that complement activation may play a region-specific role in diabetic kidney injury, highlighting the importance of monitoring C3 levels and providing a new perspective for the clinical diagnosis and treatment of this subset of patients.

In contrast, no significant association was observed between serum C3 and glomerulosclerosis, which may be attributed to the presence of potent complement regulatory proteins in the glomeruli that specifically attenuate complement-mediated injury in this region. Previous studies have indicated that glomerular injury in DKD is primarily driven by hemodynamic and metabolic pathways (17, 18). Altered renal hemodynamics can cause podocyte damage under high pressure, leading to detachment or apoptosis. This process exposes basement membrane components (e.g., laminin) and releases cellular contents (e.g., mitochondrial DNA), thereby activating the lectin pathway and progressively exacerbating microvascular injury in the kidney (19).In patients with DKD, these substances function as damage-associated molecular patterns. The impaired filtration barrier permits leakage of plasma complement components, including C3, into Bowman’s space and tubular lumina, promoting excessive complement system activation and further exacerbating localized renal injury (20).

As a core component of the complement system, complement C3 exerts dual roles of in renal injury, contributing to both injury mediation and repair regulation (21, 22). Li et al. found that C3 preferentially promotes renal interstitial, tubular and vascular damage through multiple mechanisms (23, 24). In the Ins2Akita mouse model, complement cascade proteins C3, C4b and IGHM in renal tissue are significantly increased in both early and advanced stages of DKD, indicating that complement system activation is linked to inflammation and fibrosis in DKD. Also, immunofluorescence analysis of human kidney biopsies from patients with DKD revealed increased expression of C3, C1q and IgM proteins, which are directly associated with fibrosis and inflammation (25).

Serum C3 levels are known to exhibit intra-individual variability due to factors such as fluctuations in disease activity, age, and medication use, which may influence complement synthesis, consumption, or urinary loss. Therefore, our findings should be interpreted as demonstrating an association between serum C3 levels at a single time point and renal pathological injury, rather than evidence of a dynamic process (26, 27). We observed a significant interaction between serum C3 levels and eGFR categories. In patients with lower eGFR, the association between C3 and pathological scores appears stronger, suggesting that complement activation plays a more prominent pathogenic role when renal function is impaired. We acknowledge that the absolute difference in serum C3 levels between groups was modest. However, we propose that this finding should be interpreted in the context of relative depletion rather than absolute deficiency. Even small decrements within the normal range may reflect ongoing complement consumption at the tissue level, particularly given the significant associations observed between lower serum C3 and more severe renal pathological injuries (e.g., IFTA) in the low eGFR group. This suggests that in DKD, serum C3 may function as a continuous risk marker rather than a dichotomous diagnostic test. From a clinical perspective, a single C3 measurement alone is insufficient for prognostic assessment, its integration with other markers and pathological findings may enhance risk stratification (28). These findings provide new insights for risk stratification for diabetic patients with reduced eGFR: even a mild decline in serum C3 may reflect more severe intrarenal inflammation and tissue damage, suggesting that such patients could benefit from earlier initiation of renal protective therapy or complement-targeted interventions (29).

The weak inverse correlation between serum C3 levels and renal C3 deposition observed in this study reflects the complex pathophysiology of diabetic microvascular complications. Serum C3 represents a snapshot of systemic levels, whereas renal C3 deposition indicates chronic accumulation, demonstrating a clear temporal discrepancy. Besides, local C3 production and activation, interindividual variability in renal clearance, and heterogeneity in complement activation may further weaken the relationship between systemic C3 and tissue deposition (30). Although modest, the negative correlation aligns with theoretical expectations, suggesting that C3 dose translocation from circulation to tissues, albeit under multifactorial regulation and through complex mechanisms that require further investigation. These findings further suggest that in clinical practice, serum C3 alone is an inadequate surrogate for inferring renal C3 deposition, as these two parameters may reflect distinct aspects of complement involvement in DKD. A comprehensive evaluation integrating other biomarkers and dynamic monitoring is warranted for more accurate risk stratification and pathological interpretation.

Follow-up analysis showed that patients in the lower C3 group had a significantly greater increase in 24-h proteinuria at 1-year follow-up compared with the higher C3 group. Notably, there were no significant differences between the two groups in traditional risk factors, including age, blood pressure, Alb, TC, HbA1c and baseline urinary protein levels (31, 32). Cross-sectionally, lower serum C3 levels were independently associated with more severe renal pathological injury. Exploratory longitudinal analysis suggested a potential association with proteinuria progression, but this finding requires confirmation in larger prospective studies due to limited follow-up data.

### 4.1 Limitations

Several limitations of this study should be acknowledged. First, as this is a biopsy-based cohort, selection bias is inherent. The indications for renal biopsy in diabetic patients typically select those with atypical presentations or more aggressive clinical courses. Consequently, our study population may not fully represent the entire spectrum of DKD. Moreover, Serum C3 levels were measured only once at the time of renal biopsy, which may not capture temporal variability in complement levels over the course of disease progression. The absence of systematic measurements of alternative pathway components and terminal effectors restricts comprehensive analysis of the complement cascade, requiring further in-depth investigation. Future multicenter studies with larger, consecutive cohorts, particularly those including early-stage DKD patients who do not undergo biopsy, are warranted to validate our findings.

## 5 Conclusion

This study suggests that lower serum C3 levels may serve as an immunopathological marker associated with advanced renal damage in DKD, particularly in relation to progressive histological injuries such as IFTA. Incorporating C3 measurement into the clinical management of patients with impaired renal function (eGFR < 90 mL/min/1.73m²) offers a valuable tool for improving prognostic accuracy. This strategy allows for the early identification of patients at high risk, supporting timely and targeted interventions that may ultimately improve renal outcomes.

## Data Availability

The datasets presented in this article are not readily available because due to institutional regulations and ethical constraints, the raw data underlying this study cannot be shared publicly or upon request. Requests to access the datasets should be directed to Minghao Guo, gmhdoctor@163.com.
